# A remarkable assemblage of ticks from mid-Cretaceous Burmese amber

**DOI:** 10.1017/S0031182022000269

**Published:** 2022-05

**Authors:** Lidia Chitimia-Dobler, Ben J. Mans, Stephan Handschuh, Jason A. Dunlop

**Affiliations:** 1 Bundeswehr Institute of Microbiology, Neuherbergstrasse 11, D-80937 Munich, Germany; 2 Epidemiology, Parasites and Vectors, Agricultural Research Council-Onderstepoort Veterinary Research, Onderstepoort, South Africa; 3 The Department of Veterinary Tropical Diseases, University of Pretoria, Pretoria, South Africa; 4 Department of Life and Consumer Sciences, University of South Africa, Pretoria, South Africa; 5 VetCore Facility for Research, Veterinärmedizinische Universität Wien, Veterinärplatz 1, A-1210 Vienna, Austria; 6 Museum für Naturkunde, Leibniz Institute for Evolution and Biodiversity Science, Invalidenstrasse 43, D-10115 Berlin, Germany

**Keywords:** Burmese amber, *Cornupalpatum*, *Deinocroton*, *Ixodes*, *Khimaira*, tick fossil

## Abstract

Four fossil ticks (Arachnida: Parasitiformes: Ixodida) are described from mid-Cretaceous (ca. 100 Ma) Burmese amber of Myanmar. *Ixodes antiquorum* sp. nov. (Ixodidae) is the first Mesozoic record of *Ixodes* and the oldest representative of the most species-rich extant tick genus. Its affinities appear to lie with modern Australian forms, consistent with the hypothesis that Burmese amber hosted Gondwanan faunal elements. Even more remarkable is *Khimaira fossus* gen. et sp. nov. which combines a body resembling that of a soft tick (Argasidae) with a basis capitulum more like that of a hard tick (Ixodidae). We refer it to Khimairidae fam. nov. as a possible transitional form between the two main families of ticks alive today. Another member of the extinct Deinocrotonidae is described as *Deinocroton copia* sp. nov., while the first described adult female for *Cornupalpatum burmanicum* is associated with a dinosaur feather barb.

## Graphical abstract

**Figure:**
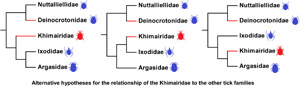

## Introduction

Ticks (Parasitiformes: Ixodida) are distinctive arachnids, all of which are haematophagous ectoparasites of vertebrates. As important vectors of several diseases in humans and livestock, they have attracted a considerable body of research (Sonenshine and Roe, 2013). Approximately 905 living species are conventionally divided into ~714 hard ticks (Ixodidae), ~190 soft ticks (Argasidae), plus a further family (Nuttalliellidae) with a single species (Beati and Klompen, 2019). Fossil ticks are rare but have occasionally been recorded as subfossils assignable to living species (Sanchez *et al*., 2010). Most tick fossils are inclusions in amber. Both hard and soft ticks are known from Miocene Dominican Republic amber (Lane and Poinar, 1986; Poinar, 1995), dated 20–15 Ma (Peris *et al*., 2015). There is a hard tick from Eocene (ca. 49–44 Ma) Baltic amber and a soft tick from Late Cretaceous (ca. 94–90 Ma) New Jersey amber (Weidner, 1964; Klompen and Grimaldi, 2001). The oldest, and most productive, source of fossil ticks is the mid-Cretaceous (ca. 100 Ma) Burmese amber of Myanmar. This deposit hosts a surprisingly diverse fauna including two extinct genera of hard ticks, *Cornupalpatum* (Poinar and Brown, 2003) and *Compluriscutula* (Poinar and Buckley, 2008), alongside fossils assigned to two living hard tick genera, *Amblyomma* Koch, 1844 and *Haemaphysalis* Koch, 1844 (Klompen in Grimaldi *et al*., 2002; Chitimia-Dobler *et al*., 2017, 2018). There is also an extinct family and genus: Deinocrotonidae and *Deinocroton* Peñalver, Arillo, Anderson and Pérez-de la Fuente, 2017 (Peñalver *et al*., 2017).

Hard ticks are further subdivided into two clades: Prostriata, containing the genus *Ixodes* Latreille, 1795 (Latreille, 1795), and Metastriata encompassing the remaining Ixodidae genera. Prostriates and metastriates can be distinguished on characters such as the position of the groove around the anus and the absence or presence of festoons around the posterior edge of the body. All hard ticks found in Burmese amber so far have been metastriates, while the oldest prostriate is an *Ixodes* species from Baltic amber (Weidner, 1964; Dunlop *et al*., 2016). Here, we describe the first *Ixodes* tick from Burmese amber doubling the stratigraphic range of Prostriata. The second inclusion represents the first adult female of *Cornupalpatum* in Burmese amber and, like a previous record (Peñalver *et al*., 2017), is associated with a dinosaur feather which has implications for its feeding ecology. A third inclusion represents a new species belonging to the previously described extinct genus *Deinocroton*. The final and most surprising inclusion is even more interesting having a body resembling that of a soft tick, but a capitulum (the region bearing the mouthparts) like that of a hard tick. This latter inclusion represents an extinct lineage, potentially ancestral to the two main tick families today. However, molecular data suggest that the split between Ixodidae and Argasidae was considerably older than the mid-Cretaceous (Mans *et al*., 2012, 2019), which could imply that the new fossil is a late survivor of an earlier radiation.

## Materials and methods

### Material

Three fossils originate from the private collection of Patrick Müller and bear the specimen numbers BUB4185 (*Ixodes antiquorum* sp. nov.), BUB4029 (*Khimaira fossus* sp. nov.), BUB3319 (*Deinocroton copia* sp. nov.). One is from the collection of Lidia Chitimia-Dobler (the *Cornupalpatum* female). Specimens from Patrick Müller have been deposited in the Paleontological collection in Munich (BUB4185: SNSB-BSPG 2021 XII 10; BUB4029: SNSB-BSPG 2021 XII 11; BUB3319: SNSB-BSPG 2021 XII 12) and from Lidia Chitimia-Dobler in the Museum für Naturkunde Berlin.

### Imaging

For photography, a Keyence VHX-7000 Digital Microscope with an FI 4K Revolver Head (Keyence Itasca, IL, USA), an X-Y and Z-motorized stage, and a tiltable stand, with a combination of incident and transmitted light for focus stacking and a Keyence VHX-900F (Keyence Itasca), were used. Magnifications ranged from 100 to 1000 times. Polarized light was used for some images to resolve more details, and the resulting image stacks were combined using the software Helicon Focus 6.7.1. Microscopic computed tomography (microCT) scans were acquired using a Zeiss XRadia MicroXCT-400 (Carl Zeiss X-ray Microscopy, Pleasanton, CA, USA). Acquisition settings were adapted depending on the size of the specimen, size of the amber piece and required level of detail. For the *D. copia* sp. nov. specimen, the whole body was scanned at 80 kVp per 100 *μ*A with 30 s exposure using the 0.4× detector assembly resulting in 4.64 *μ*m isotropic voxel size. For the *K. fossus* sp. nov. specimen, the whole body was scanned at 80 kVp per 100 *μ*A with 30 s exposure using the 4× detector assembly resulting in 1.95 *μ*m isotropic voxel size. For the *I. antiquorum* sp. nov. specimen, capitulum and scutum were scanned at 80 kVp per 50 *μ*A with 60 s exposure using the 20× detector assembly resulting in 0.44 *μ*m isotropic voxel size. For the *Cornupalpatum* female, the whole body was scanned at 40 kVp per 200 *μ*A with 30 s exposure using the 4× detector assembly resulting in 1.37 *μ*m isotropic voxel size. All scans were recorded over a 360° specimen rotation with an angular increment of 0.225° between projections. Image volumes were processed and visualized by volume rendering using the 3D software package Amira 6.4. Drawings were prepared with a *camera lucida* attachment on a Leica M205C stereomicroscope (Leica Microsystems, Wetzlar, Germany), again using a combination of incident and transmitted light where appropriate.

## Results

### A new prostriate fossil

Class Arachnida Lamarck, 1801

Order Parasitiformes Reuter, 1909

Suborder Ixodida Leach, 1815

Family Ixodidae Murray, 1877

*Ixodes* Latreille, 1795

*Ixodes antiquorum* Chitimia-Dobler, Mans and Dunlop sp. nov.

*Etymology.* From the Latin *antiquus* (aged, ancient).

**Fig. 1.**
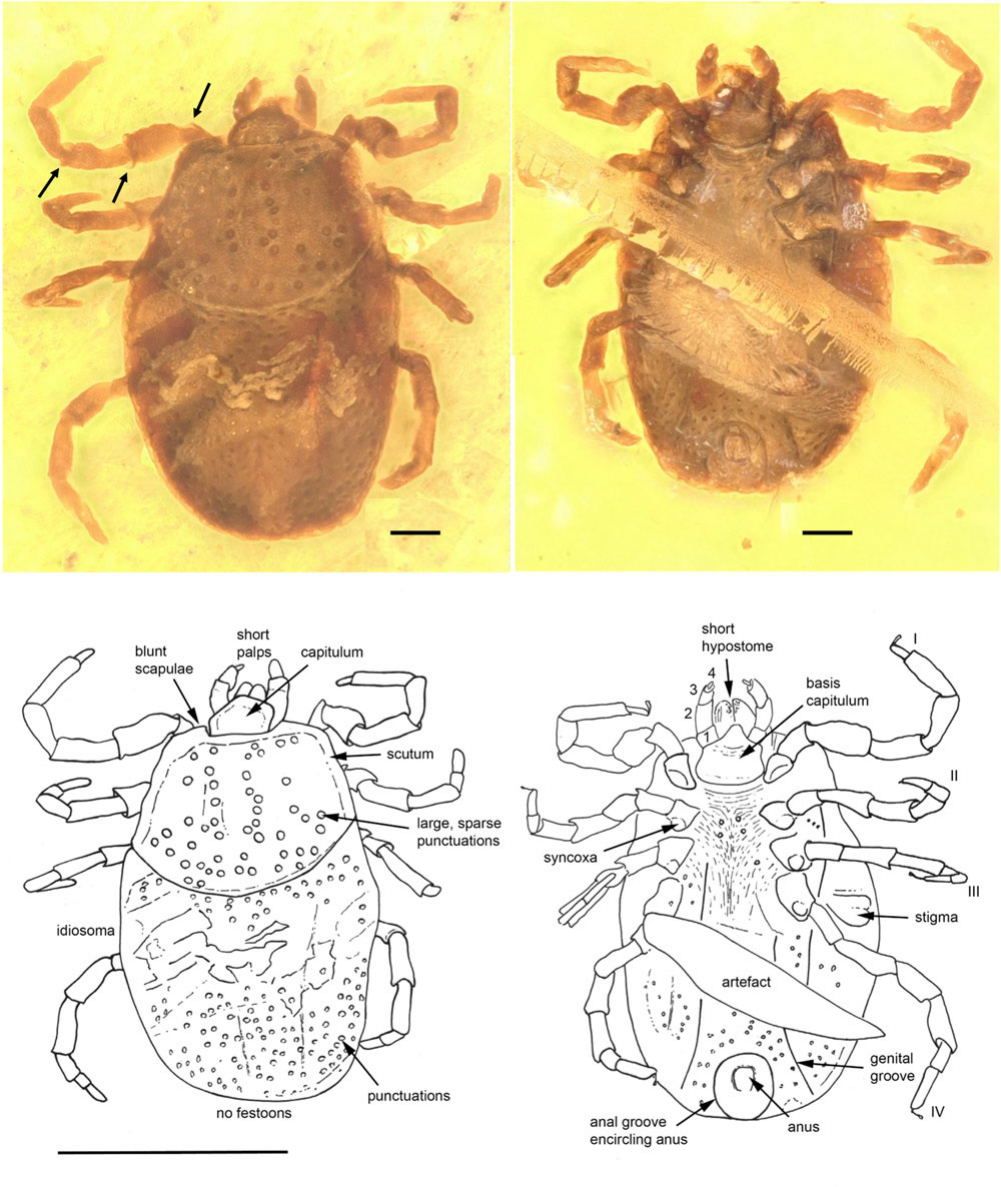
*Ixodes antiquorum* sp. nov. (Ixodidae) from Burmese amber designated as the holotype for this species. Indicated are dorsal (left) and ventral (right) images. The absence of festoons and the anterior anal groove can be clearly discerned. Arrows indicate the presence of notch-like processes on the joints. Line drawings at the bottom indicate important aspects described in the text. Scale bars indicated are 0.1 mm for the photos and 1 mm for line drawings.

*Holotype*. Female tick (BUB4185) (Fig. 1) deposited in the Munich Paleontological Collection. The species name was registered with Zoobank (LSID code: zoobank.org:act:739BD930-5C1C-4E6E-8C4A-A05180B216A8).

*Diagnosis*. *Ixodes* nymphal tick in which the anal groove encircles the anus anteriorly, eyes absent, festoons absent, coxae without spurs. Scutum wider than long, carinae absent on basis capituli and scutum; trochanter, femur and genu articulations with notch-like processes.

### Description of nymph

*Idiosoma*: body ovoid, length from middle of scutum to posterior body margin: 0.793 mm; maximum width (measured in middle, behind third legs) 0.565 mm; dorsal and ventral surface without setae, but with a moderate number of large punctuations (Fig. 1). Scutum 0.471 mm wide (measured in middle) and 0.342 mm long (from middle to edge); broadest prior to the posterior end, large sparse punctuations distributed throughout scutum, sides straight and diverging posteriorly, posterior margin slightly convex, posterior corrugations absent; scapulae blunt (Fig. 1). Cervical grooves not visible (Fig. 1). Anus visible, median; anal groove encircling anus anteriorly and converging posteriorly (Fig. 1). Genital groove visible only at posteroventral edge of idiosoma. Stigma subtriangular with small macula (transverse axis 0.120 mm by 0.059 mm) behind right IV coxa.

*Capitulum*: Length from apices to the posterior margin of basis 0.134 mm. Basis capituli almost rectangular dorsally with posterolateral margins a little divergent anteriorly; posterior margin straight, cornuae absent; ventrally rectangular, posterior margin rounded, length from palpal insertion to the posterior margin of basis 0.064 mm, width 0.151 mm, no auriculae. Palpi short, thick, convex dorsally, much separated at the base, with long axes converging in front; four articles with lengths: trochanter 1, 0.023 mm; femur 2, 0.059 mm; genu 3, 0.040 mm; tibiotarsus 4, 0.017 mm. Hypostome short, bluntly rounded apically, 0.096 mm in length, denticles arranged in 3–4 rows from top to bottom. More detail on the file and number of denticles was not available due to the presence of host-derived tissue on the hypostome. Chelicerae well developed, equal in length to hypostome.

*Legs*: Coxae subtriangular, internal and external spurs absent, syncoxae present on all coxae. Tarsus I gradually stepped and tarsi II–IV stepped. Trochanter, femur and genu joints of all legs have notch-like processes, and spurs dorsally and ventrally (Fig. 1).

*Chaetotaxy*: Two setae observed on all leg articles, and four setae associated with Haller's organ. Palps bear small setae on the femur and 4–5 small setae on genu proximate to the joint with the tibiotarsus.

*Remarks*. *Ixodes* are currently subdivided into 16 subgenera (Clifford *et al*., 1973; Robbins and Keirans, 1992; Durden and Keirans, 1996). Our new fossil species cannot be placed with confidence in any particular living subgenus as it possesses morphological features consistent with a number of different taxa. The fossil shares a number of morphological aspects with the members of the subgenus *Endopalpiger* and *Exopalpiger*: broader than longer scutum with sparse large punctuations (not dense as in *Ixodes tasmani* Neumann, 1899), blunt scapulae, scutum carinae and cornua absent, and the anal groove and coxae are quite similar. Morphological characters shared with nymphs of *Ixodes holocyclus* Neumann, 1899 (*Sternalixodes*) include the trochanter small, round and somewhat salient laterally but visible only ventrally. The sternal plate is absent in the nymph but can be present in females (Durden and Keirans, 1996). The presence of syncoxae in the fossil is a morphological character observed in adults of some species from the subgenus *Endopalpiger* and in adults and nymphs of some *Sternalixodes* species (Roberts, 1960). These ticks possess a type of scutum, broad posteriorly, which appears to be somewhat characteristic of Australian forms. It is observable in *Ixodes australiensis* Neumann, 1908, *Ixodes ornithorhynchi* Lucas, 1845 and *I*. *tasmani*, and the scutum of the nymph of *Ixodes vestitus* Neumann, 1908 is of this shape (Roberts, 1960). Like the fossil, some Australian living *Ixodes* species cannot be easily placed in a subgenus, such as *Ixodes barkeri* Barker, 2019; *Ixodes heathi* Kwak, Madden and Wicker, 2018; *Ixodes woylie* Ash *et al*., 2017; and *Ixodes laridis* Heath and Palma, 2017 (Barker, 2019) based on morphological features.

The hypostome of the fossil could not be described in detail due to a piece of soft tissue from the host that is still attached to this structure (Supplementary Fig. 1). This is the first observation of soft tissue still attached to the hypostome of a fossil tick. The presence of an artefact identified as a possible mammalian hair (Fig. 1) is also of interest and is suggestive of a possible host for this tick species.

### The first female fossil for *Cornupalpatum*

Family Ixodidae Murray, 1877

*Cornupalpatum burmanicum* Poinar and Brown, 2003

### Description of unengorged adult female

**Fig. 2.**
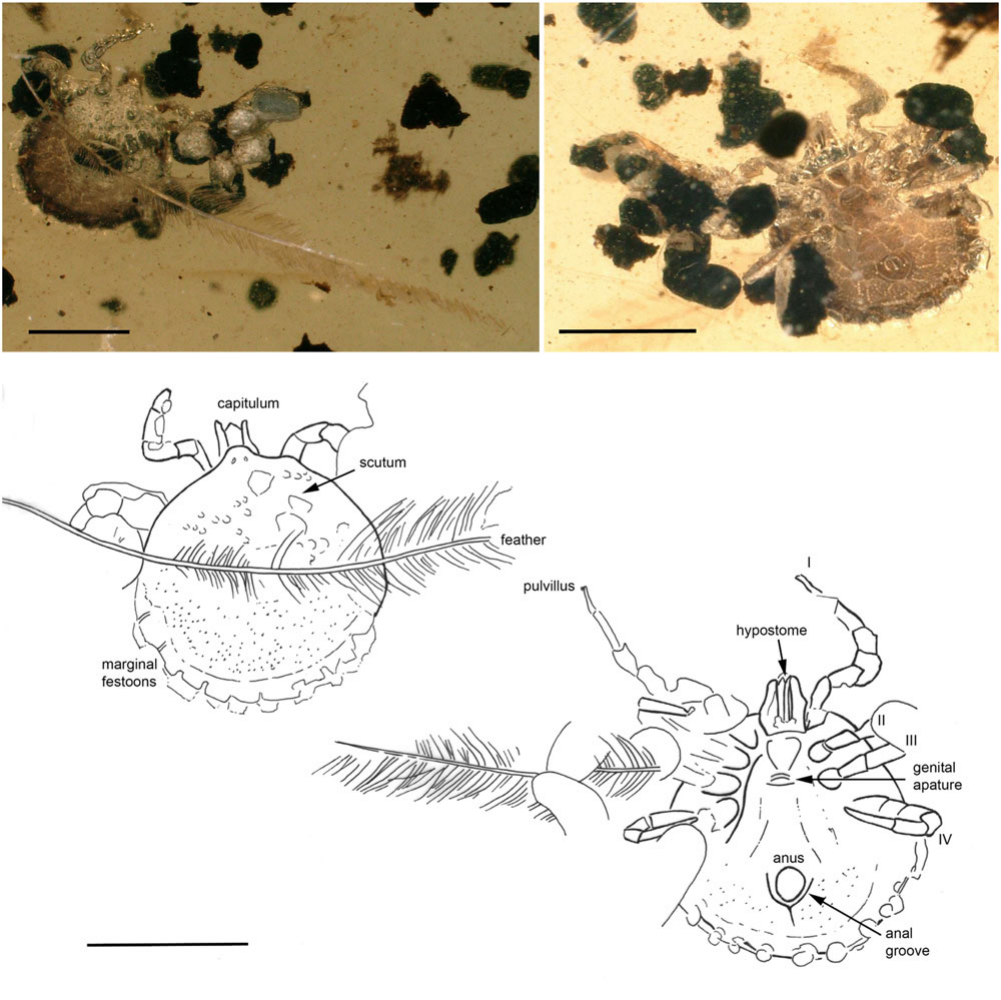
A fossil of a female *Cornupalpatum burmanicum* (Ixodidae). Indicated are dorsal (up) and ventral (down images). The genital aperture, anus and the posterior V anal groove can be clearly discerned. A dinosaurian feather can be seen on the dorsal side. A line drawing at the bottom indicates important features. The scale bar in all figures is 1 mm.

*Idiosoma*: Ornamentation indistinct; body subcircular, length from middle of scutum to posterior body margin: 1.392 mm; maximum width (measured in middle, behind third legs) 1.435 mm; dorsal and ventral surface without setae, but with moderate number of small punctuations (Fig. 2). Scutum can be seen only on the posterior part and seems to be subtriangular (Fig. 2). Eleven festoons. Anus visible, median; anal groove behind the anus, well visible, large ‘V’ shape (Fig. 2). Genital aperture median, forming transverse slit with the edges twisted inward, like a loop, situated between coxae III; spiracle plates comma-shaped, medial and lateral margins parallel, dorsal prolongation long, broad, perpendicular to the anterior–posterior axis, macula, round, situated subterminally; genital groove absent.

*Capitulum*: Length from apices to the posterior margin of basis 0.388 mm; basis capituli posterior margin straight ventrally, hooks on the internal sides of the genu; hypostome length 0.258 mm, columns of teeth on hypostome are 2/2 blunt-tipped teeth, with internal line 6 teeth and external line with 7 teeth; apical end like a wide blade with well-developed lateral hooks oriented anteroposteriorly (Supplementary Fig. 2).

*Legs*: Coxae I–IV with no obvious spurs; tarsus I tapering distally, clear, oval area on the tarsi I dorsum is Haller's organ; claws paired, slender, simple, slightly curved; with distinct pulvillus visible on some legs.

*Chaetotaxy*: small setae visible on some legs joint and tarsi I; long setae on the third palpal segment and around the Haller's organ were observed.

*Remarks*. The present specimen is the first adult female of this species and, like a previous record by Peñalver *et al*. (2017), it is associated with a feather. On the dorsal side of the tick is the barb of a pennaceous feather. It is 5.903 mm long. Parts of the many barbules are broken; nevertheless, on the distal part of some barbules, hooklets can be seen. The barbules share similar morphology in their attaching base. Distal ramus and the barbules from one side are not visible, probably damaged before having become embedded in the resin. One of the claws on the first leg of the tick fossil grasps a barb from another feather (Supplementary Fig. 3). This provides further support for the hypothesis that *C. burmanicum* used feathered dinosaurs as hosts (Peñalver *et al*., 2017): both as an immature tick and now potentially as an adult female.

### A new *Deinocroton* species

Family Deinocrotonidae Peñalver, Arillo, Anderson and Pérez-de la Fuente, 2017

*Deinocroton* Peñalver, Arillo, Anderson and Pérez-de la Fuente, 2017

*Deinocroton copia* Chitimia-Dobler, Mans and Dunlop sp. nov.

*Etymology*. From the Latin *copia* (abundance) to describe the apparent species abundance of this genus in the Myanmar amber deposits.

**Fig. 3.**
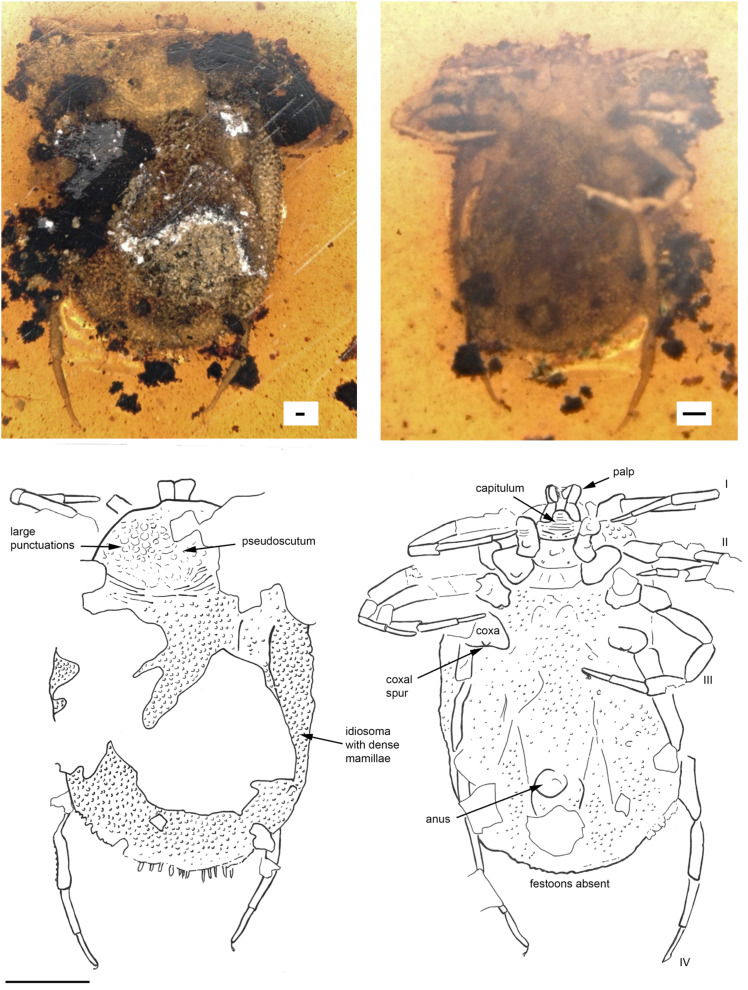
*Deinocroton copia* sp. nov. (Deinocrotonidae) in Burmese amber designated as the holotype for this species. Indicated are dorsal and ventral views. Line drawings indicate pertinent features. The scale bars for the photos are 0.1 mm and for the line drawing 1 mm.

*Holotype*. Female (BUB3319) (Fig. 3) deposited in the Munich Paleontological Collection. The species name was registered with Zoobank (LSID code: zoobank.org:act:FEB6E4CC-BE4F-4764-9E23-98F41505DE43).

*Diagnosis*. Female *D. copia* sp. nov. possess on coxae I a single median spur, coxae II two spurs, medial posterior and distal anterior, and only a single small anterior spur on coxae III and a small median blind spur on coxae IV. Genital aperture between coxae II.

### Description of female

*Idiosoma*: body subcircular; length from middle of pseudoscutum to posterior body margin: 4.018 mm; dorsal and ventral surface with dense mammillae, without discs or sutural line between dorsal and ventral surface (Fig. 3). Pseudoscutum in anterodorsal view is posteriorly broadened, 1.346 mm wide (measured in middle) and 1.063 mm long (from middle to edge); with large punctuations on the lateral sides, cervical grooves absent (Fig. 3); genital aperture between coxae II; anus and spiracles not visible. Eyes and festoons absent.

*Capitulum*: Capitulum not visible in dorsal view, rectangular ventral 0.180 mm wide and 0.441 long, boarded by the coxae I; hypostome subterminal not well visible; trochanter short and robust, femur longest, distally thickened in width and height, and with a blade-like formation in the middle of the internal side, genu bent ventral direction (creating a ventral concavity, with the surface of the femur) from the joint with the femur, wide and with spinous and transverse processes, tibiotarsus shorter than the femur, sword-like (Fig. 3, Supplementary Video).

*Legs*: Coxae well developed; coxae I and IV with a median spur, coxae II with two spurs; coxae III with small external spur; trochanter, femur, genu and tarsi articulations with notch-like processes. Dorsal and ventral edges of femur, genu, tibia and tarsi riffled (Supplementary Fig. 4).

*Chaetotaxy*: No visible setae.

*Remarks*: Characters that differentiate *D. copia* sp. nov. from *D. draculi* derive mainly from the number and locality of the spurs found on the coxae. For coxae I, both species possess a single median spur. For coxae II, *D. copia* possesses two spurs, medial posterior and distal anterior, while *D. draculi* possesses three spurs, two medial and one distal anterior. For coxae III and IV, *D. draculi* presents three spurs, two basal and posterior, and one medial anterior. *Deinocroton copia* sp. nov. only presents a single small anterior spur on coxae III and a small median blind spur on coxae IV. Genital aperture between coxae II in *D. copia* and between coxae II and coxae III in *D. draculi*.

### A new tick family

Family Khimairidae Chitimia-Dobler, Mans and Dunlop fam. nov.

This family name was registered with Zoobank (LSID code: zoobank.org:act:E8C0A1D8-1364-46D9-A052-33E700E4FEE8).

*Diagnosis*. Nymphs with soft body, terminal gnathostoma, dense mammillae on body surface, discs on main body absent, scutum present, sutural line between dorsal and ventral surface and tarsal dorsal humps absent, pulvillus poorly developed.

*Khimaira* Chitimia-Dobler, Mans and Dunlop gen. nov.

The genus name was registered with Zoobank (LSID code: zoobank.org:act:EE3B8A7C-4470-4D25-9A4C-3ECF01DAB19C).

*Etymology*. From the ancient Greek khímaira (**χῐ́μαιρᾰ**), a mythological animal combining parts of more than one creature.

*Diagnosis*. As for the family.

*Khimaira fossus* Chitimia-Dobler, Mans and Dunlop gen. et sp. nov.

*Etymology*. From the Latin fossō (dig), as in a fossil.

**Fig. 4.**
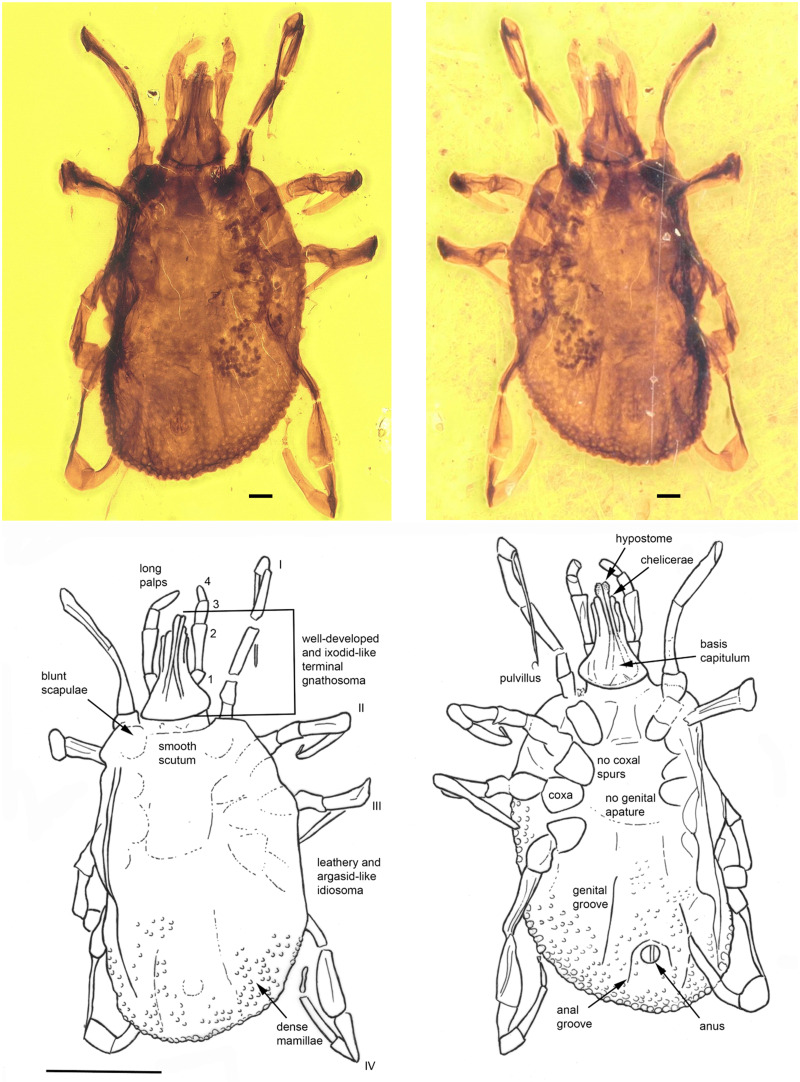
*Khimaira fossus* gen. et sp. nov. (Khimairidae) in Burmese amber designated as the holotype for this species and the family Khimairida. The terminal gnathostoma, scutum and mammillated alloscutum can be clearly discerned. The line drawing indicates pertinent features. The scale bars for the photos are 0.1 mm and for the line drawing 1 mm.

*Holotype*. Nymph (BUB4029) (Fig. 4) deposited in the Munich Paleontological Collection. The species name was registered with Zoobank (LSID code: zoobank.org:act:BED4E24E-8477-4810-9259-F693037FB37D).

*Diagnosis*. As for the family.

### Description of nymph

*Idiosoma*: body oval, length from middle of scutum to posterior body margin: 1.277 mm; dorsal and ventral surface with dense mammillae, without discs or sutural line between dorsal and ventral surface (Fig. 4). Scutum subtriangular, 0.703 mm wide (measured in middle) and 0.522 mm long (from middle to edge); scapulae blunt (Fig. 4). Anus visible, median on the posterior part of idiosoma; anal groove slightly visible, encircling the anus anteriorly closing the sides above the idiosoma edge (Fig. 4). Stigmas located between coxae III and IV, broadly oval, longer axis transverse 0.155 mm × 0.095 mm. Genital aperture absent; genital groove visible posteriorly, on the side of anal groove. Eyes and festoons absent.

*Capitulum*: Length from apices to the posterior margin of basis 0.534 mm. Basis capituli outline roughly triangular, length from palpal insertion to the posterior margin of basis 0.127 mm, width 0.298 mm, no auriculae. Palpi long; four articles with lengths: trochanter, 0.074 mm; femur, 0.200 mm; genu, 0.093 mm; tibiotarsus, 0.115 mm. Hypostome arising from a flared anterior extension of the basal ‘collar’ of the capitulum, extending to below the level of chelicera distal end and the anterior third of the femur; apex bluntly pointed; dental formula 2/2; chelicera shorter than hypostome.

*Legs*: long, slender; coxae generally narrow, elongate oval, without spurs; tarsi gradually stepped, without humps; claws long, slender, simple, pulvilli poorly developed (Fig. 4).

*Chaetotaxy*: No visible setae.

*Remarks*. This fossil is interpreted as a nymph as it has four pairs of legs, but no genital aperture or porose areas which are specific characters for adult females. It is not a male as it presents a smooth scutum only on the anterior part of the idiosoma. The idiosoma has a leathery cuticle composed of innumerable small mammillae and lacks a lateral sutural line, thus resembling the cuticle of living *Ornithodoros* ticks in the family Argasidae. The intermammillary space and discs being absent further render it similar to nymphs of the *Ornithodoros moubata* group (Bakkes *et al*., 2018). The stigmata that are located between coxae III and IV are also similar to soft ticks, compared to hard ticks where the stigmata are located behind coxae IV.

Despite the similarities to the Ornithodorinae, a remarkable feature of *K. fossus* gen. et sp. nov. is the fact the gnathosoma is in a terminal position; a character otherwise only seen in ixodid ticks. The gnathosoma of the new species is well-developed and has the second article of palps two times longer than articles 1 and 3. This is specifically seen in extant ixodids belonging to the genus *Amblyomma* (Nicholson *et al*., 2009). The fossil also has a scutum, a feature unique to hard ticks although it is difficult to determine whether the composition of the scutum is sclerotized as observed in hard ticks, or whether it is closer to the semi-sclerotized pseudo-scutum observed for *N. namaqua* and *D. draculi* (Latif *et al*., 2012; Peñalver *et al*., 2017). The capitulum of the new fossil has an extended collar around the chelicera and hypostome, similar to larvae of *Carios quadridentatus* (Heath, 2012). The chelicerae are partly visible and seem to have an outer and inner digitus and are shorter than the hypostome of *N. namaqua* (Latif *et al*., 2012). The mammillated character of the integument differs from that of the Deinocrotonidae and Nuttalliellidae which both present a wrinkled integument with closely spaced pits. Taken together, an argasid-like body with ixodid-like mouthparts represents a unique combination of characters which merit a new, extinct family.

## Discussion

Burmese amber originates from the Hukawng Valley in the Kachin State of northern Myanmar. It has been interpreted as a tropical forest environment (Grimaldi *et al*., 2002) and is usually dated to the mid-Cretaceous, probably Upper Albian to Lower Cenomanian, or about 100 Ma (Shi *et al*., 2012; Smith and Ross, 2018). Burmese amber hosts a rich fauna (Ross, 2019, 2020), predominantly terrestrial arthropods. Debate remains about the precise palaeogeographic position of the locality during the time of amber deposition (Poinar, 2018), which impacts on the question to what extent the flora and fauna had their origins in Gondwana or Laurasia. Westerweel *et al*. (2019) suggested that the Burma terrane was an island within the Trans Tethyian Arc during the mid-Cretaceous. In detail, the Burma terrane forms part of the Incertus Arc that formed ca.155 Ma and was linked to northern Australia and India *via* the Woyla Arc (Hall, 2012). This connection could have provided a short window of land bridges for colonization by Gondwanan elements before the land bridges were destroyed at ca.140 Ma (Hall, 2012). Continued northward movement would then place the Burma terrane in the Trans Tethyian Arc by the time of Burmese amber deposition at ca.100 Ma.

Burmese amber hosts the oldest known ticks, as well as the oldest records of two other members of the wider Parasitiformes clade to which the ticks belong: Opilioacarida (Dunlop and Bernardi, 2014) and Mesostigmata (Joharchi *et al*., 2021). Given that most arachnids have a fossil record going back to the Palaeozoic, the relatively young (Cretaceous) age of the oldest parasitiform mites is probably an artefact of a lack of appropriate fossil localities for preserving animals of this nature, given that many modern parasitiforms are soil organisms which are less likely to end up in lacustrine environments where they could be buried by sediment. That said, Burmese amber ticks retain their importance by offering (a) the oldest calibration points to date for molecular phylogenies of several living genera, and (b) for demonstrating that during the mid-Cretaceous the tick fauna of the amber forest included what appear to be both modern and extinct genera living side by side. It may be noted that an undescribed immature tick from Spanish amber would push the oldest tick fossils to 105 Ma (Peñalver *et al*., 2017).

With almost 250 living species, *Ixodes* is the most diverse modern tick genus and contains several species of medical importance, such as the paralysis tick *I. holocyclus* in Australia and the Lyme disease vectors *Ixodes ricinus* in the Palearctic and *Ixodes scapularis* in the Nearctic (Padula *et al*., 2020; Gilbert, 2021). *Ixodes antiquorum* sp. nov. is the oldest record of *Ixodes*, predating the Baltic amber species (Weidner, 1964) by more than 50 million years. A further putative (non-amber) *Ixodes* from the Eocene of Wyoming in the USA is not demonstrably a tick (Dunlop, 2011). A record of the extant species *Ixodes sigelos* Keirans, Clifford and Corwin, 1976 from a Holocene owl pellet in Argentina (Sanchez *et al*., 2010) is the only other unequivocal (sub)fossil in this genus. The presence of *Ixodes*, and its concomitant clade Prostriata, was to be expected in Burmese amber based on the presence of several genera from its sister-group Metastriata. According to current molecular dating, the split between Prostriata and Metastriata probably occurred considerably earlier at ca. 234 ± 18 Ma (Mans *et al*., 2019).

*Ixodes* is a cosmopolitan genus today, occurring on several continents (Clifford *et al*., 1973; Fukunaga *et al*., 2000). As noted above, *I. antiquorum* sp. nov. appears to be most closely related to modern Australian species. This is interesting for two reasons. First, it has long been recognized that there is a fundamental difference between Australian *Ixodes* species and all the other *Ixodes*, such that the Australian taxa cluster together phylogenetically and some authors even questioned the monophyly of the genus (Fukunaga *et al*., 2000; Klompen *et al*., 2000, 2007; Shao *et al*., 2005). Other studies support a monophyletic *Ixodes* (Charrier *et al*., 2019), but the fact remains that there is a deep division between the Australian and non-Australian taxa, with molecular dating suggesting a split at 224 ± 18 Ma (Mans *et al*., 2019). The fact that the Burmese amber nymph has Australasian affinities is thus interesting in anchoring this lineage to at least 100 Ma; the non-Australian *Ixodes* lineage is anchored on the Baltic amber fossil to ca. 49 Ma.

*Ixodes antiquorum* sp. nov. is also of considerable biogeographical interest and supports the hypothesis that the flora and fauna of Burmese amber have, at least in part, Gondwanan origins (Poinar, 2018). We hypothesize that the ancestors of our new species could have originated in Australia and then migrated onto the Burma Terrane about 155 Ma *via* the Woyla Arc (Hall, 2012) (see also above) before rafting north towards Asia on this terrane. The hypothesis that Burmese amber fossils may have Gondwanan affinities is not new and has been previously discussed (Hall, 2012; Yamamoto *et al*., 2019). Possible Gondwanan origins have been proposed for, e.g. several beetles (Kirejtshuk and Poinar, 2006, 2013; Cognato and Grimaldi, 2009; Thayer *et al*., 2012; Cai and Huang, 2017; Jałoszyński *et al*., 2017; Jarzembowski *et al*., 2017; Cai *et al*., 2018, 2019; Wu et al., 2018; Yamamoto *et al*., 2019) and bugs (Poinar and Brown, 2016).

An alternative hypothesis is that the West Burma block rifted from Australia in the Early-Middle Permian (~270 Ma) and was attached to Asia by the upper Triassic (~200 Ma) (Sevastjanova *et al*., 2016; Metcalfe, 2017; Clarke *et al*., 2019). The fossil species found in Burmese amber would, therefore, have been endemic to the Asiatic region by the time of fossilization ~100 Ma later. If the affinities of *I. antiquorum* sp. nov. to extant Australian *Ixodes* lineages have a monophyletic origin, this scenario would suggest a much more ancient origin for *Ixodes* (>270 Ma) and by implication ticks in general. However, it would not explain the extant restriction of Australasian *Ixodes* since subsequent dispersal on mainland Asia from 200 Ma would have suggested a much wider distribution for the Australasian *Ixodes*, given that the lineage leading to the Burmese fossils would have survived in Asia for more than 100 million years. Tick fossils may therefore have important implications for hypotheses on the origin and timing of the West Burma block.

The *C. burmanicum* female together with a feather barb completes the finding of this tick in amber from larvae (Poinar and Brown, 2003), a nymph with dinosaur feather (Peñalver *et al*., 2017), and the female from the current study. The barb from a feather found corresponds to a dinosaur feather according to Carroll *et al*. (2019). For *C. burmanicum*, the hooks on the internal side of the third palpal segment in all described stages confirm that these ticks belong to the same species. The finding of two different life stages with dinosaur feathers supports the Peñalver *et al*. (2017) hypothesis that this tick was a parasite of the Pennaraptora clade of dinosaurs.

While the presence of fossils referable to extant tick genera in Burmese amber points to a considerable degree of evolutionary stasis in some lineages (*Amblyomma*, *Haemaphysalis* and now also *Ixodes*), the discovery of *K. fossus* gen. et sp. nov. that combines the features of both hard and soft ticks is of considerable interest and importance. There is precedence for ticks in the mid-Cretaceous having body plans, unlike species that we know today. *Deinocroton* was placed in an extinct family, Deinocrotonidae (Peñalver *et al*., 2017), and differs from living species in the ornamentation of its integument, the shape of the palp and the shapes of the preanal and genital grooves. It may be related to the living family Nuttalliellidae with its single species, *Nuttalliella namaqua* Bedford, 1931, being termed a ‘living fossil’ since it presents intermediate characters between hard and soft ticks (Bedford, 1931; Mans *et al*., 2011). In detail, it has an argasid-like body, argasid-like feeding behaviour, but an ixodid-like pseudoscutum and a sub-terminal hypostome (Mans *et al*., 2012). *Deinocroton* also preserves these pseudoscutum and hypostome characters (Peñalver *et al*., 2017).

*Khimaira fossus* gen. et sp. nov. is neither a nuttalliellid nor a deinocrotonid. It has a soft, argasid-like body combined with a well-developed, ixodid-like terminal basis capitulum and a scutum. The basis capituli differs completely from the mouthparts of both Deinocrotonidae and Nuttalliellidae which is underdeveloped compared to the Khimairidae. These features, in combination, are so distinct and incongruous with respect to the known families (living and extinct) that we believe the new fossil merits its own family (Khimairidae fam. nov.) since it seems to be a truly chimaeric fusion of a hard and soft tick. This makes it a much likelier candidate than either deinocrotonids or nuttalliellids of being a last common ancestral lineage to the two main living tick families. With regard to its biology, the soft body suggests that both nymphal and adult stages would have exhibited rapid feeding behaviour, as observed for living Argasidae and Nuttalliellidae (Mans *et al*., 2016). By contrast, the terminal gnathosoma would imply that larvae may have undergone prolonged feeding, as observed in some members of Argasidae and all Ixodidae and Nuttalliellidae. This suggests that, as in the Nuttalliellidae, the ancestral biology of ticks is represented by larvae that showed prolonged feeding, with nymphs and adults showing rapid feeding (Mans *et al*., 2016). As such, Khimairidae, like Nuttalliellidae, presents characters shared among argasids and ixodids and may explain the striking differences in the biology of the two main tick families through sub-functionalization after they diverged from one another.

*Khimaira fossus* gen. et sp. nov. is mid-Cretaceous in age, but the Ixodidae/Argasidae split must predate the Prostriata/Metastriata one (see above), with published mitochondrial gene molecular dates for the origins of the two main families ranging in the literature from the Early to Late Permian: 290 ± 23 to 260 ± 21 Ma (Mans *et al*., 2012, 2019). In this scheme, all the major tick lineages found in Burmese amber originated well in advance of the formation of the Incertus Arc at 155 Ma. These include *Ixodes* (224 ± 18 Ma), *Amblyomma* (144 ± 12 Ma) and *Haemaphysalis* (173 ± 14 Ma) (Mans *et al*., 2019), which would have allowed dispersal and occupation of the Burma terrane by these lineages from Australia. Based on this scenario, Gondwanan lineages that also originated before the formation of the Incertus Arc might also be expected in Burmese amber. This would include Bothriocrotoninae (180 ± 15 Ma) as well as Argasidae (223 ± 20 Ma); the latter potentially supported by the presence of an ornithodorid tick in New Jersey amber (Klompen and Grimaldi, 2001). The presence of Bothriocrotoninae in Burmese amber would offer particularly strong support for the colonization of the Incertus Arc from Australia. Conversely, the presence of Deinocrotonidae and Khimairidae in Australian amber deposits (if these could be discovered) would be further support of this hypothesis.

A much younger origination date for ticks has been postulated in the Jurassic ~192 ± 50 Ma (Beati and Klompen, 2019), with the split between Ixodidae and Argasidae at ~178 ± 50 Ma. However, in this molecular dating based on the nuclear 18S rRNA gene, the prostriates originate at ~112 ± 50 Ma, metastriates at ~97 ± 12 Ma, Amblyomminae at ~71 ± 25 Ma and Haemaphysalinae at ~33 ± 25 Ma (Beati and Klompen, 2019). Given that fossils assignable to *Ixodes* (this study), *Amblyomma* and *Haemaphysalis* have already been found in 100 Ma Burmese amber (Chitimia-Dobler *et al*., 2017, 2018), the nuclear clock seems to underestimate divergence times for ticks, which suggests in turn that the origin of ticks may actually lie somewhat earlier, perhaps between 273 and 192 Ma (i.e. mid-Permian to Early Jurassic). Again this underscores the importance of Burmese amber tick fossils for our understanding of tick origins and evolution.

**Fig. 5.**
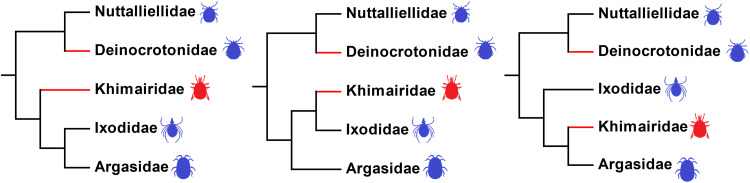
A representation of the possible systematic relationships among living and extinct tick families adapted from Peñalver *et al*. (2017) and Mans *et al*. (2019). Extinct lineages are depicted by red branches, while the potential divergence points for the Khimairidae are indicated as sister lineage to the Ixodidae/Argasidae (preferred placement), or the Ixodidae, or the Argasidae.

Given its relatively young age, our new fossil is unlikely to be directly ancestral to either of the modern families (Fig. 5). Instead, we suspect there was a late Palaeozoic, or perhaps early Mesozoic, lineage from which both Ixodidae and Argasidae evolved and that *K. fossus* gen. et sp. nov. is part of this group and retained these intermediate character states through into the late Mesozoic. A similar scenario from Burmese amber was observed in the remarkable tailed spider *Chimerarachne yingae* (Wang *et al*., 2018). This extinct species retains several plesiomorphic character states for spiders (Huang *et al*., 2018; Wang *et al*., 2018) most obviously the retention of a flagelliform telson, but cannot be directly ancestral to other Araneae as spiders referable to the extant clade Mesothelae were already present in the Late Carboniferous. We hypothesize that the mid-Cretaceous Burmese amber hosted late survivors of earlier radiations of at least the ticks and spiders among the arachnids, and it would be interesting to see if this is true of any other arachnid groups. Examples of unusual Burmese amber insects with character combinations not seen in living groups are also known (Bai *et al*., 2016; Poinar and Brown, 2017), and may represent further examples of relict arthropod taxa which maintained a presence until near the end of the Mesozoic – at least on the putative island hosting the Burmese amber forest.

## Data Availability

The data reported in this paper are detailed in the main text.

## Supplementary material

For supplementary material accompanying this paper visit https://doi.org/10.1017/S0031182022000269. click here to view supplementary material
