# Supplementary material for: A remarkable assemblage of ticks from mid-Cretaceous Burmese amber
Source: Parasitology. 2022 Mar 4;149(6):820–30. doi: 10.1017/S0031182022000269 (PMC10090602; doi:10.1017/S0031182022000269)
Supplement: Supplementary file 1 [file S0031182022000269sup.zip › S0031182022000269sup001.docx]

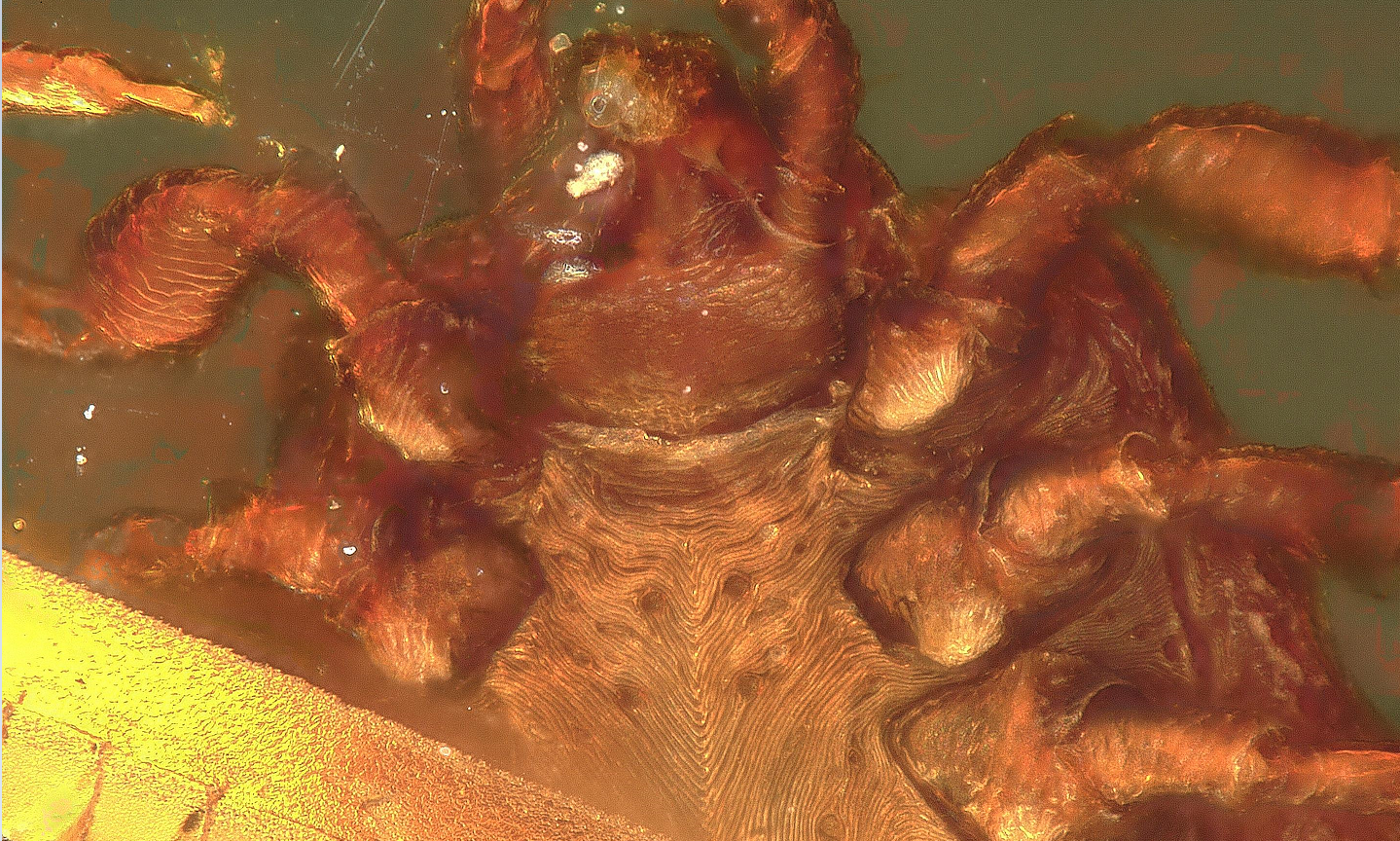


**Supplementary figure 1:** Ventral close-up of the mouth parts of *Ixodes* *antiquorum*. The tissue attached to mouth parts can be observed as a opaque inclusion.

**
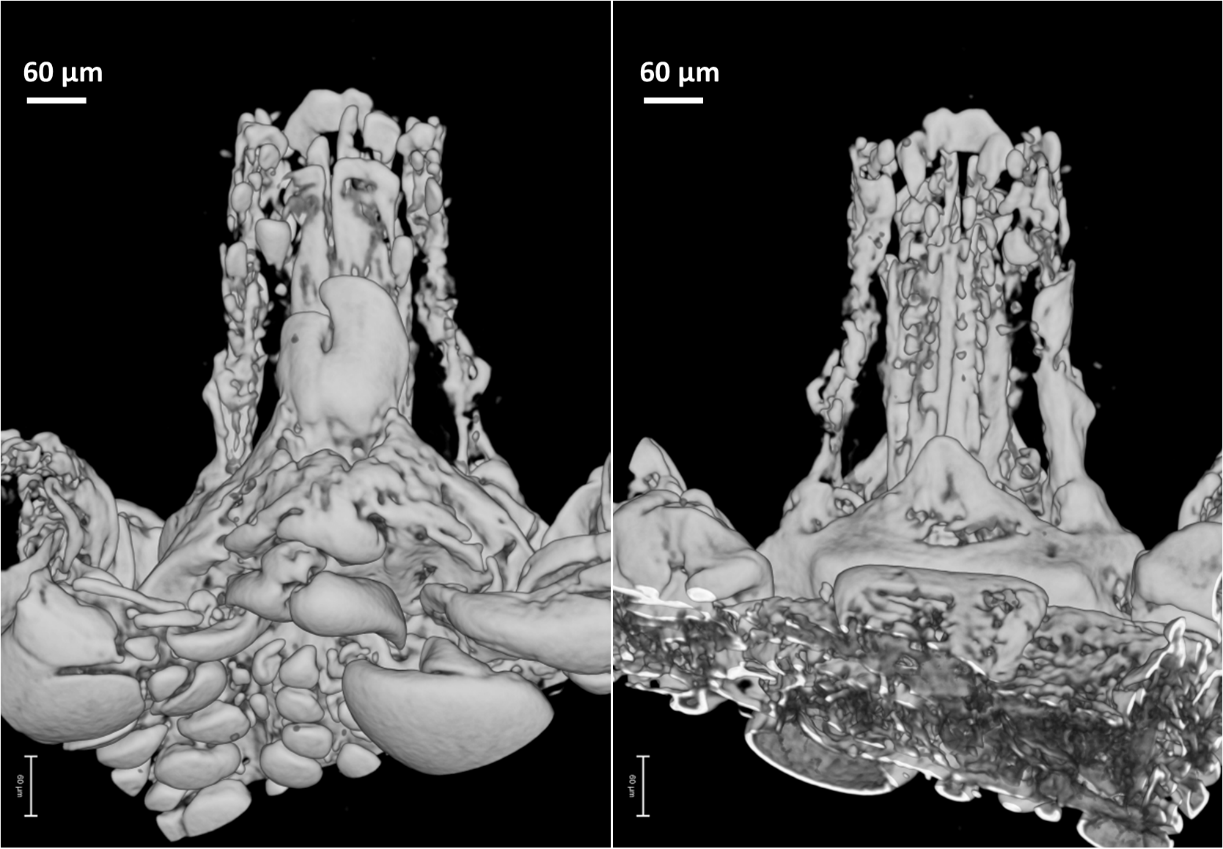
**

**Supplementary figure 2:** MicroCT scan of the hypostome of *Cornupalpatum burmanicum*. Indicated is the dorsal view on the left and the ventral view on the right.


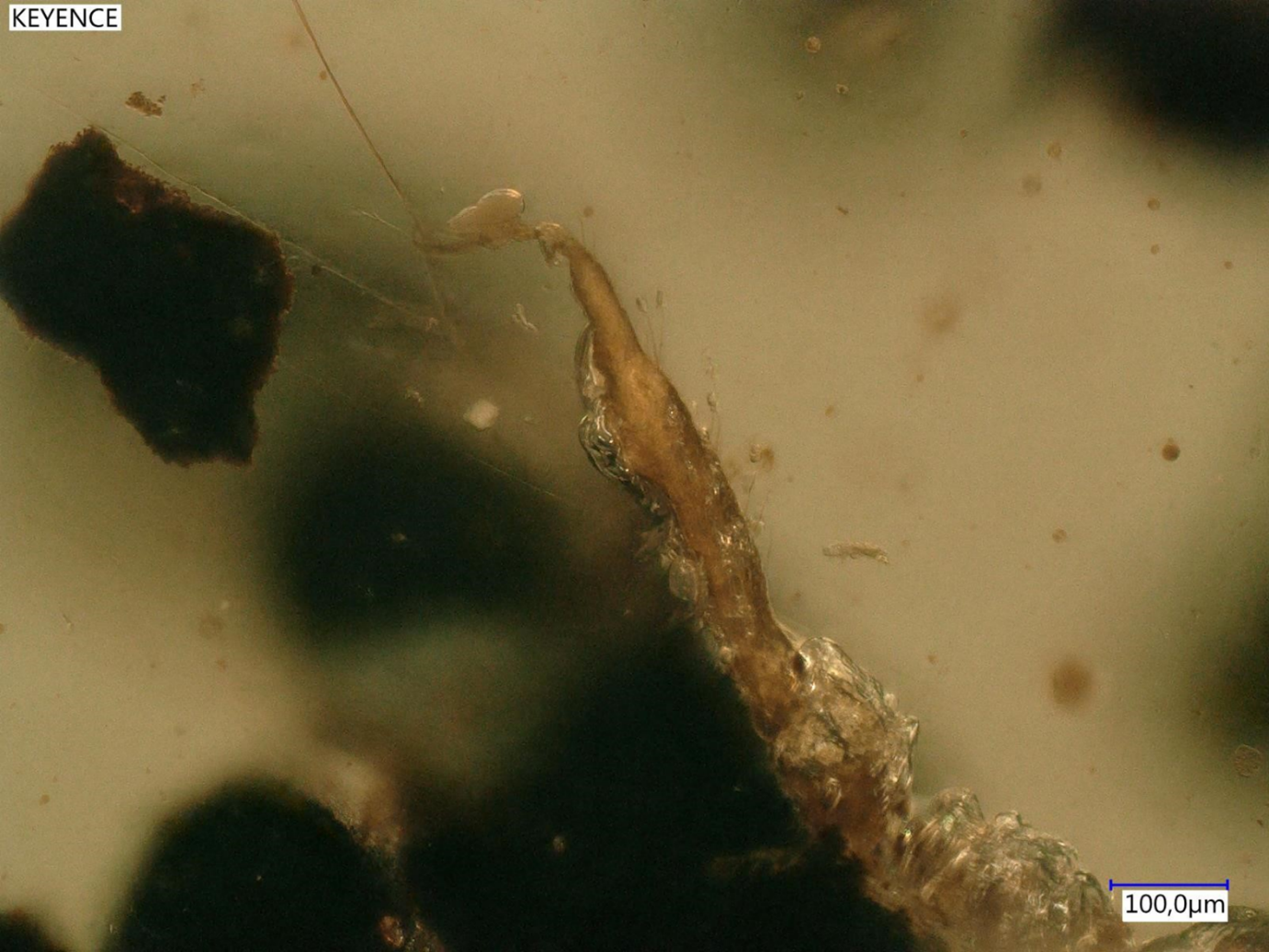


**Supplementary figure 3:** Tarsi from *Cornupalpatum burmanicum* grasping a barb from a feather.


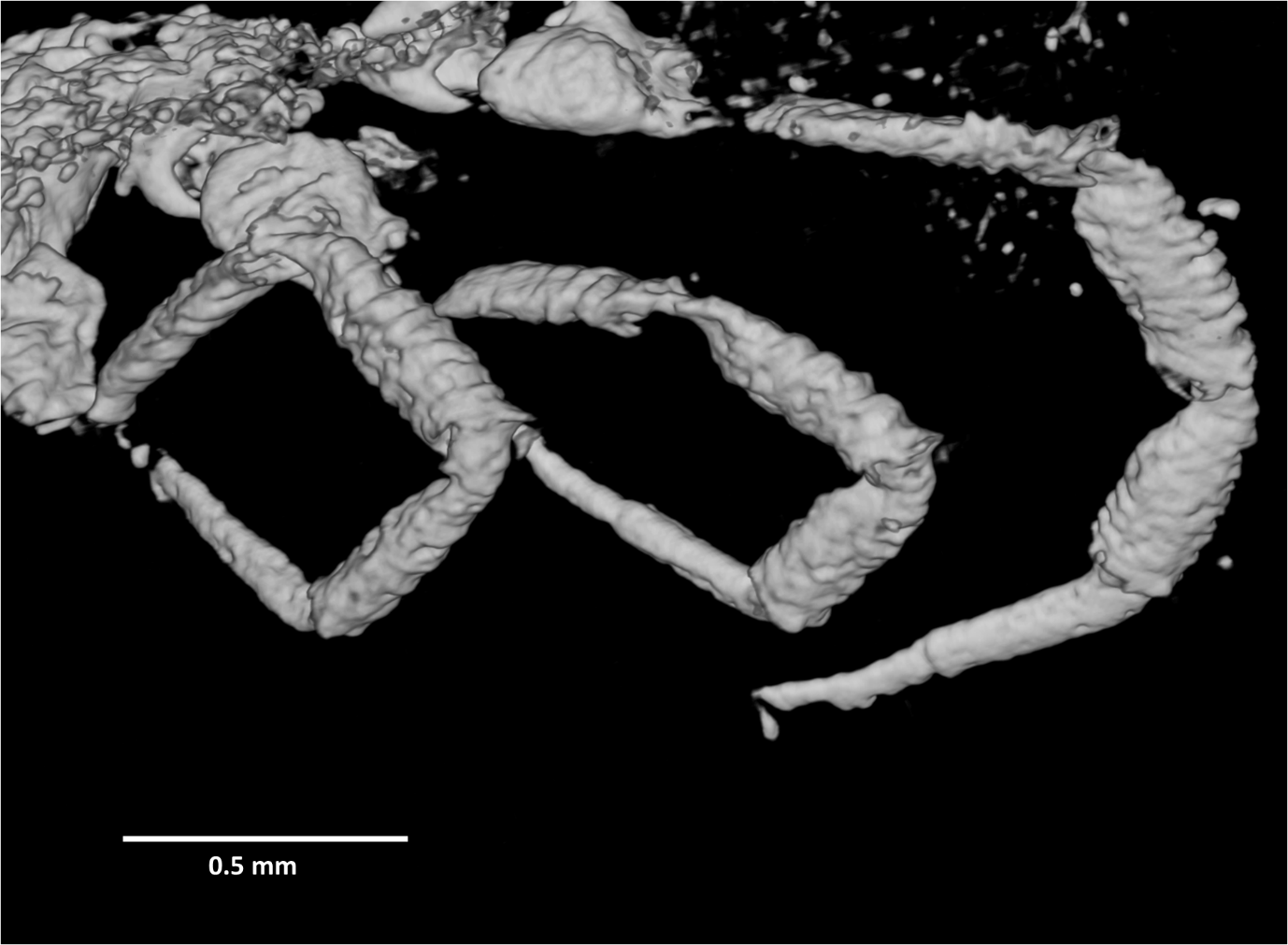


**Supplementary figure 4:** MicroCT scan of the legs of *Deinocroton copia* sp. nov. The tarsi articulations with notch-like processes and riffled dorsal and ventral line can also be observed.

**Supplementary video:** A rendering of the microCT scan of the palps of *Deinocroton copia* sp. nov.
